# Photosynthetic gas exchange, plant water relations and osmotic adjustment of three tropical perennials during drought stress and re-watering

**DOI:** 10.1371/journal.pone.0298908

**Published:** 2024-02-28

**Authors:** Jie He, Klaudia Ng, Lin Qin, Yuanjie Shen, Harianto Rahardjo, Chien Looi Wang, Huiling Kew, Yong Chuan Chua, Choon Hock Poh, Subhadip Ghosh

**Affiliations:** 1 Natural Sciences and Science Education Academic Group, National Institute of Education, Nanyang Technological University, Singapore, Singapore; 2 Nanyang Technological University, School of Civil and Environmental Engineering, Singapore, Singapore; 3 Housing & Development Board, Building & Research Institute, Singapore, Singapore; 4 Plant Science & Health and Centre for Urban Greenery and Ecology, National Parks Board Headquarters, Singapore, Singapore; United Arab Emirates University, UNITED ARAB EMIRATES

## Abstract

Planting vegetation on slopes is an effective way of improving slope stability while enhancing the aesthetic appearance of the landscape. However, plants growing on slopes are susceptible to natural drought stress (DS) conditions which commonly lead to water deficit in plant tissues that affect plant health and growth. This study investigated the photosynthetic gas exchange, plant water status and proline accumulation of three tropical perennials namely *Clerodendrum paniculatum*, *Ipomoea pes-caprae* and *Melastoma malabathricum* after being subjected to DS and re-watering (RW). During DS, there was a significant decrease in light-saturated photosynthetic CO_2_ assimilation rate (*A*_*sat*_), stomatal conductance (*g*_*s sat*_), and transpiration rate (*T*_*r*_) for all three plant species. Leaf relative water content, shoot water potential, and leaf, stem and root water content also declined during DS. Proline concentration increased for all three species during DS, reaching especially high levels for *C*. *paniculatum*, suggesting that it heavily relies on the accumulation of proline to cope with DS. Most of the parameters recovered almost completely to levels similar to well-watered plants after RW, apart from *M*. *malabathricum*. Strong linear correlations were found between *A*_*sat*_ and *g*_*s sat*_ and between *g*_*s sat*_ and *T*_*r*_. Ultimately, *C*. *paniculatum* and *I*. *pes-caprae* had better drought tolerance than *M*. *malabathricum*.

## Introduction

In recent decades, rising global temperatures have generated rapid climate changes, together with more intense rainfall events and longer droughts [1]. Consequently, these changes result in adverse effects on the moisture content of soils. Moisture content in soil is the key to plant health and slope stability. An intense rainfall event may cause a slope failure or a tree failure, resulting in damage to public infrastructures and endangering public safety [2, 3]. On the other hand, drought is one of the most significant abiotic stressors that limit plant growth. Large regions of the tropics experience seasonal droughts every year. Global climate change has also contributed to increased erratic weather patterns such as escalated frequencies, intensities and prolonging of drought [4, 5]. Under drought stress (DS) conditions, water deficit normally develops in plant tissues. Changes in plant water status are a direct consequence of DS. These changes are generally observed later than stomata responses. Plant water deficit can be measured by both tissue (leaf, stem and root) water content and leaf relative water content (RWC). Tissue water content was determined by desiccation to constant weight while leaf RWC reflects the degree of water saturation in leaves, indicating the balance between water supply to the leaf and transpiration rate [6].

Plants have various complex mechanisms to deal with water loss, for example, morphological and physiological acclimatisation at a plant-level, and the involvement of drought-tolerant genes and proteins at a molecular level [7]. These mechanisms are used to achieve drought avoidance, escape and ultimately tolerance if the drought condition persists. At the plant level, a key physiological process known to initiate photosynthetic response to DS is the closure of stomata [8]. This directly reduces water loss to the environment via transpiration which also limits the diffusion of CO_2_ from the atmosphere to the plant and then to the chloroplasts, thereby slowing photosynthetic gas exchange. Since there is a linear relationship between stomatal conductance (*g*_*s*_) and photosynthetic CO_2_ demand [9], a decline in *g*_*s*_ during stomatal closure would result in a decrease in net CO_2_ assimilation rate (*A*). A study by Maseda and Fernandex [10] demonstrated that the rapid closure of the stomata during DS caused a decrease in *A* and transpiration rate (*T*_*r*_). The trade-off between closing the stomata to reduce water loss and keeping it open to assimilate CO_2_ for photosynthesis varies for plant different species. Stomatal closure also regulates water potential in response to DS. According to the literature, plant water use strategies can be divided into isohydric and anisohydric [11]. Plants known as isohydric plants close their stomata early on in drought conditions to minimize water losses and avoid excessively low leaf water potential. However, this results in the suppression of their photosynthetic carbon gain from the beginning of the DS [12]. Conversely, anisohydric plants tend to be not as strict with stomatal regulation, using a more wasteful water use strategy to achieve higher productivity [13].

Other than adjustment of stomata aperture, plants can achieve increased drought tolerance through osmotic regulation by the accumulation of solutes. This can include the accumulation of osmolytes such as amino acids like proline, and soluble sugars. These solutes are usually products of nitrogen and/or carbon metabolism. Accumulating solutes decreases cell osmotic potential, causing water to enter the cell and the cells can maintain their turgor [14]. Additionally, having these solutes in the cell cytoplasm also helps to protect important enzymes in photosynthesis and allows for plant growth amid environmental stressors [15].

Singapore is reported to have erratic and unpredictable weather yearly, where thundery showers have been occurring frequently [16]. Extreme changes in weather conditions, such as dry spells followed by intense rainfall can result in mini landslides occurring. Plants make good ground covers and growing perennials on open slopes can significantly increase the strength of soil [17–19]. This helps to reduce incidences of landslides around the island as vegetation has been utilized for slope stabilization for many centuries [20]. A meta-analysis by Eziz et al. [21] highlighted that the roots of herbaceous plants were more susceptible to drought than shrubs, while the leaf mass fractions of woody plants were relatively more affected by drought than herbaceous plants. Thus, a herbaceous creeper such as *Ipomoea pes-caprae* and two woody shrubs namely *Clerodendrum paniculatum*, and *Melastoma malabathricum* commonly grown on slopes in Singapore were used in this study. The main objectives of this study are to investigate 1) the effects of drought stress (DS) and re-watering (RW) on photosynthetic gas exchange, plant water relations and osmotic adjustment; and 2) if there are any relationships among photosynthetic gas exchange, water relations and osmotic adjustment during DS and RW. To achieve these objectives, the following parameters were investigated: 1) light-saturated photosynthetic CO_2_ assimilation rate (*A*_*sat*_); 2) stomatal conductance (*g*_*s sat*_); 3) transpiration rate (*T*_*r*_); 4) leaf relative water content (RWC) and shoot water potential (ψ_shoot_); 5) leaf, stem and root water content and 6) leaf proline concentration. Drought stress is predicted to be more widespread and extreme in the near future [22]. The results of this study could provide important physiological traits for understanding how plants respond to extreme drought, which can help identify plants with greater resistance to DS and predict the response of tropical perennials grown on natural slopes to future climate change.

## Materials and methods

### Acclimatization of plants

The three tropical perennial plants *C*. *paniculatum*, *I*. *pes-caprae* and *M*. *malabathricum* were purchased from a local nursery in Singapore, which were cultivated in the same nursery in Malaysia. Upon delivery, similar sizes of healthy plants for each species with simlar number of leaves were selected for replanting. *C*. *paniculatum* and *M*. *malabathricum* were replanted in pots (30cm x 20cm, diameter x height) while *I*. *pes-caprae* was translanted into troughs (64cm x 20cm x 8cm, length x width x height) with the approved soil mixture (loam soil: compost: washed sand = 3:2:1). All pots or troughs contained the same amount of soil. The plants were well spaced out and grown in a greenhouse at the National Institute of Education and were allowed to acclimatise for 12 days there before DS and RW treatments. The maximal photosynthetic photon flux density (PPFD) inside the greenhouse was 600 to 800 μmol m^-2^ s^-1^ during midday on sunny days. The ambient temperature and relative humidity ranged from 24°C to 39°C and 38% to 98.2%, respectively.

### Drought stress (DS) and re-watering (RW) treatments

**Fig 1:**
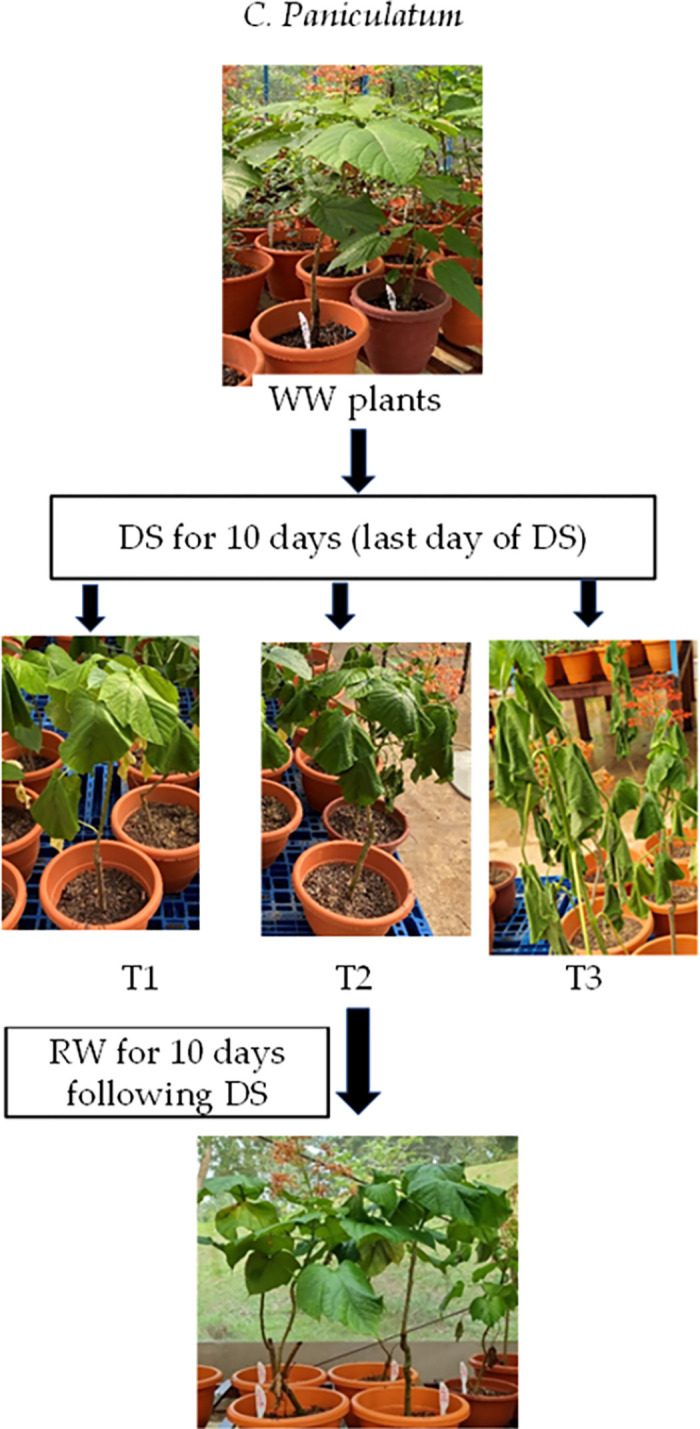
Well-watered (WW) *C*. *paniculatum* plants after drought stress (DS) for 10 days (T1, T2 and T3) and re-watering (RW) following DS for 10 days (only shows a T2 plant after RW for 10 days). WW, well-watered; T1, mild stress; T2, intermediate stress; T3, severe stress.

**Fig 2:**
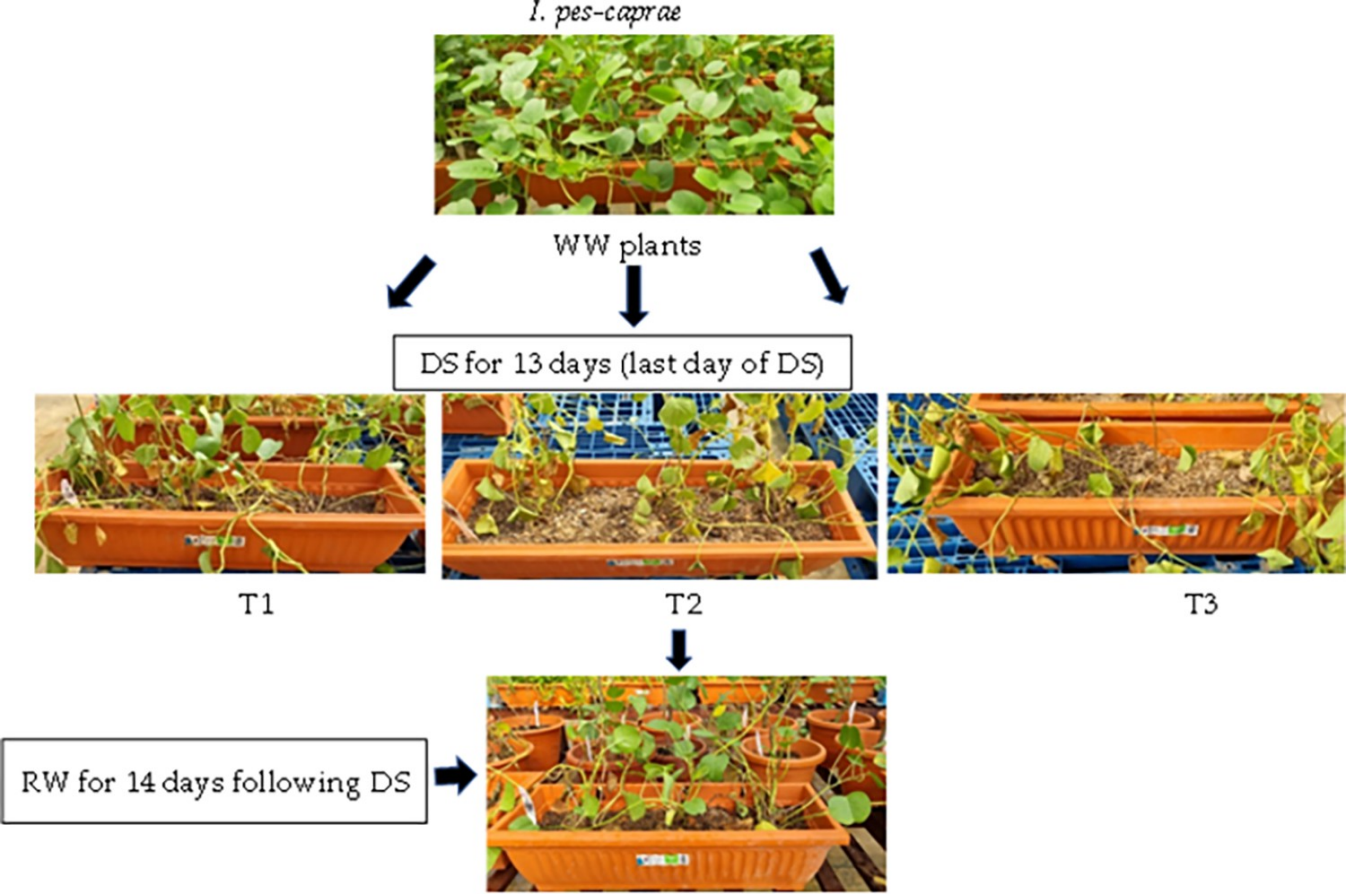
Well-watered (WW) *I*. *pes-caprae* plants after drought stress (DS) for 13 days (T1, T2 and T3) and re-watering (RW) following DS for 14 days (only shows T2 plants after RW for 14 days). WW, well-watered; T1, mild stress; T2, intermediate stress; T3, severe stress.

**Fig 3:**
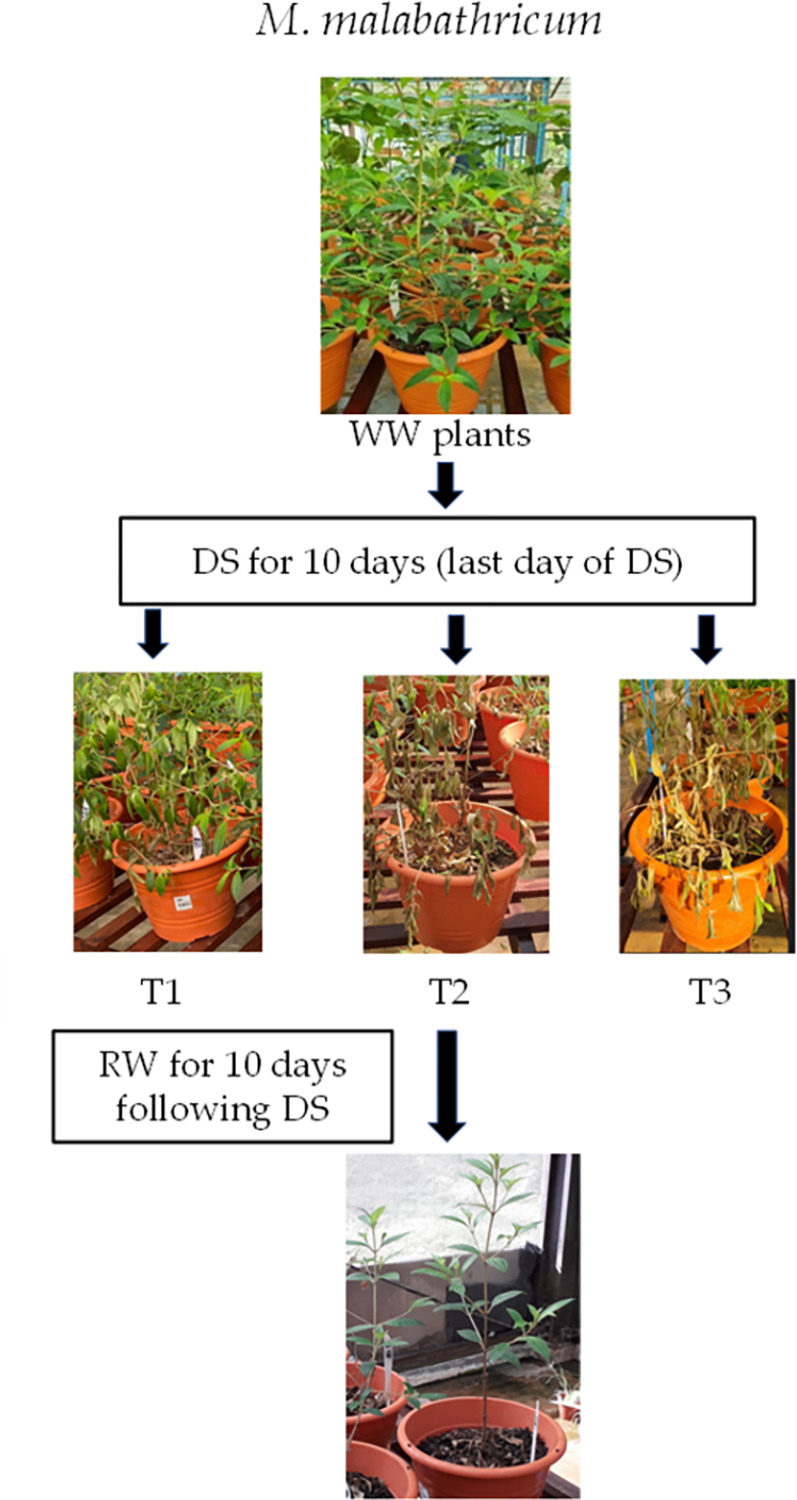
Well-watered (WW) *M*. *malabathricum* plants after drought stress (DS) for 10 days (T1, T2 and T3) and re-watering (RW) following DS for 10 days (only shows T2 plants after RW for 10 days). WW, well-watered; T1, mild stress; T2, intermediate stress; T3, severe stress.

Initially, plants were well watered (WW) to field capacity, which is the amount of soil water content held in soil after excess water has drained away, twice daily at 0700 h and 1800 h. When the drought treatments began, some plants for each species stopped receiving irrigation (defined as DS plants) while the remaining plants continued to be watered (defined as WW plants). These WW and DS plants were arranged separately in the greenhouse due to the different irrigation regimes. The DS plants were divided into three different conditions of increasing degrees of stress–mild (defined as T1), intermediate (defined as T2), and severe (defined as T3). This categorization depended on their morphological appearance such as leaf dryness symptoms, the maximal quantum efficiency of PSII, chlorophyll fluorescence F_v_/F_m_ ratio (measured by another team simultaneously under the same project) and their leaf photosynthetic parameters measured using LI-6400 portable photosynthesis system (LICOR, Biosciences, US). Within each species, the DS plants showed varying dryness symptoms after the same duration of DS treatment, which is likely due to some variations of light intensity (±80 μmol m^-2^ s^-1^ during midday on sunny days) at different positions of the greenhouse. The plants were also harvested in batches periodically to measure their water status, and leaf proline using the methods mentioned later in this section. After 10 or 13 days of DS treatment, when the plants showed great extents of stress–e.g., severe wilting, greatly reduced F_v_/F_m_ ratios and very low photosynthetic rate, the plants were rewatered twice daily to field capacity and then harvested periodically to measure tissue water content. C. *paniculatum* and *M*. *malabathricum* were rewatered following 10 days of DS treatment, while *I*. *pes-caprae* was rewatered after 13 days of DS treatment due to their different susceptibility to DS treatment. Figs 1–3 illustrate the details of DS and RW treatments for each of the three different plant species.

### Measurements of photosynthetic gas exchange

For each treatment, the light-saturated photosynthetic CO_2_ assimilation rate (*A*_*sat*_), stomatal conductance (*g*_*s sat*_), and transpiration rate (*T*_*r*_) of attached young fully expanded leaves were measured simultaneously between 10.00 h to 11.00 h inside the greenhouse using a LI-6400 portable photosynthesis system (LICOR, Biosciences, US). The readings were measured under an LED light source supplying a saturated PPFD of 1000 μmol∙m^-2^∙s^-1^ with wavelengths ranging from 420 to 510 nm and 610 to 730 nm. Average ambient [CO_2_] and relative humidity in the chamber were 400 ± 5 μmol mol^-1^ and 70%, respectively. Measurements were recorded when *A*_*sat*_, *g*_*s sat*_, and *T*_*r*_ were stable. For every treatment, four measurements were taken from four different leaves (n = 4).

### Determinations of leaf, stem and root water status

Leaf relative water content (RWC) was measured by punching three to four leaf discs (1cm diameter) from each plant after harvesting and immediately weighing their fresh weight (FW). The leaf discs were left floating in water in the dark for 24 hours, and then their saturated weight (SW) was measured. Lastly, their dry weight (DW) was obtained by drying the leaf disks in an oven at 80°C for 72 h. Leaf RWC was estimated as RWC = (FW−DW)/(SW−DW) Ο100%. Leaf water content (LWC), stem water content (StWC), and root water content (RtWC) were measured after harvesting whole plants during the DS and RW treatments. After each harvest, leaves, stems and roots were separated, and their FW were recorded. The plant parts were wrapped in a pre-weighed aluminium foil, allowed to dry at 80°C for at least five days, and then re-weighed to find their DW. The LWC, StWC and RtWC were estimated as (FW−DW)/FW X 100%. Lastly, shoot water potential (ψ_shoot_) of the harvested plants was measured using a pressure chamber (PMS Instrument Co., Corvallis, Oregon USA). The shoots were first severed using a scalpel, then quickly sealed in a pressure chamber with the severed end protruding out. The pressure chamber was pressurized with nitrogen gas until the xylem sap emerged from the cut end of the shoot. At this point, the positive pressure required to force xylem sap exudates from the tissue (displayed by the pressure gauge) is equal to the negative water potential of the xylem.

### Determination of leaf proline concentration

The details of extraction and assay for proline were described by He et al. [23]. Leaf material (0.5 g) was grounded with 3% sulfosalicylic acid (6 ml) before centrifuging at 9000 rpm for 20 min at 4°C. The supernatant (1 ml) was then mixed with equal volumes of acid-ninhydrin and acetic acid before placing it into a 95°C water bath for 1 h. To extract proline, 2 ml of toluene was added, and the mixture was vortexed for 30 seconds. The absorbance was read at 520 nm using a spectrophotometer (UV-2550 spectrophotometer, Shimadzu, Kyoto, Japan).

### Statistical analysis

One-way analysis of variance (ANOVA) was carried out to determine if there were any statistical differences between treatment groups. If the p-value was less than 0.05, a post-hoc Tukey’s Honest Significant Difference (HSD) test was used to find the pair(s) of treatment groups which were significantly different. The statistical analyses were carried out on Microsoft Excel using the ‘Real statistics’ data analysis add-in tool.

## Results

### Responses of photosynthetic gas exchange to DS and RW treatments

**Fig 4:**
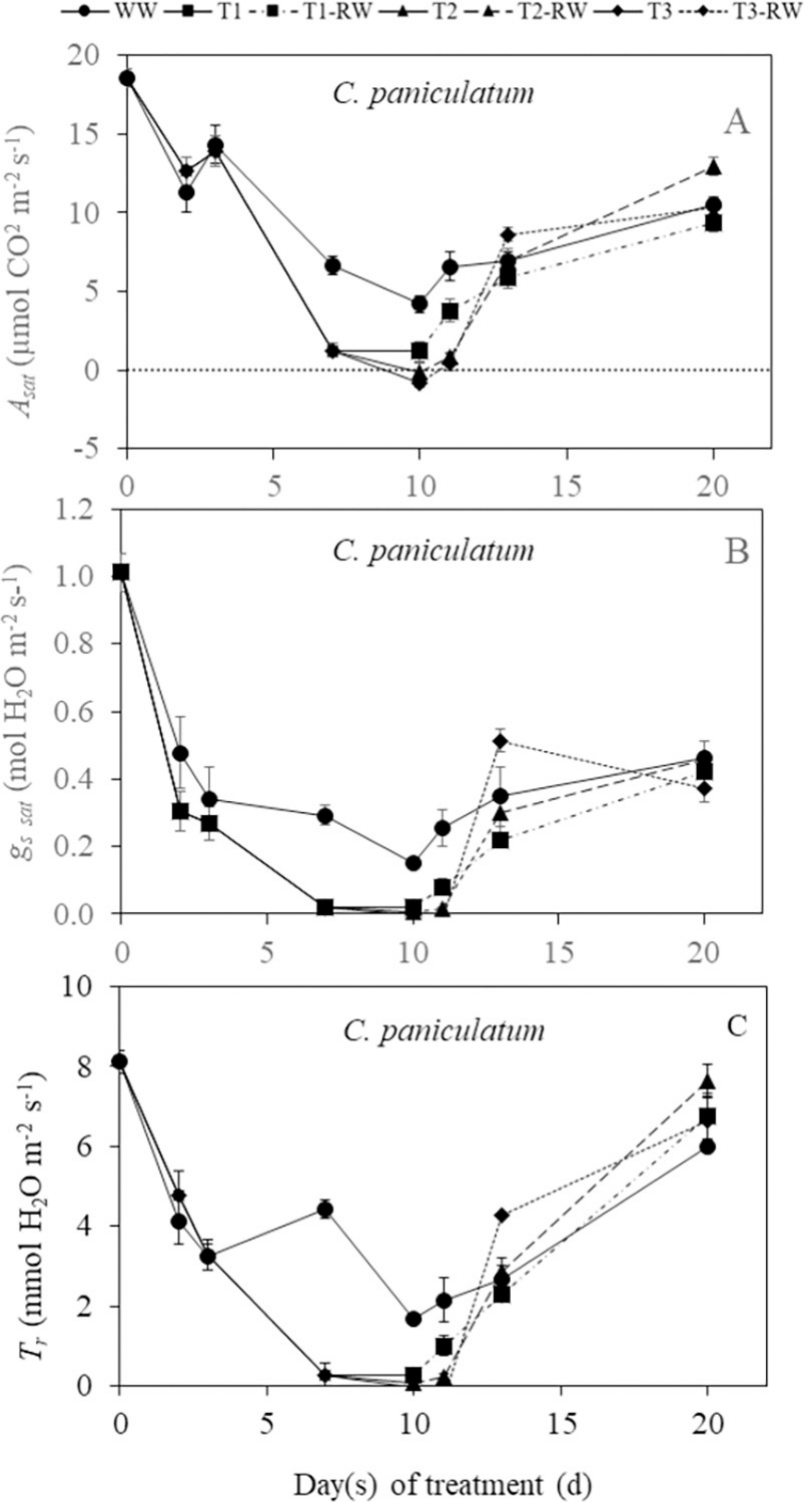
Changes in light-saturated photosynthetic CO_2_ assimilation rate, *A*_*sat*_, (A), stomatal conductance, *g*_*s sat*_ (B) and transpiration rate, *T*_*r*_ (C) of *C*. *paniculatum* during drought stress (DS) and re-watering (RW) treatments. Values are means (± S.E.) (n = 4). Any significant differences (p<0.05) are indicated in the text due to limited space in the figure. WW, well-watered; T1, mild stress; T2, intermediate stress; T3, severe stress.

**Fig 5:**
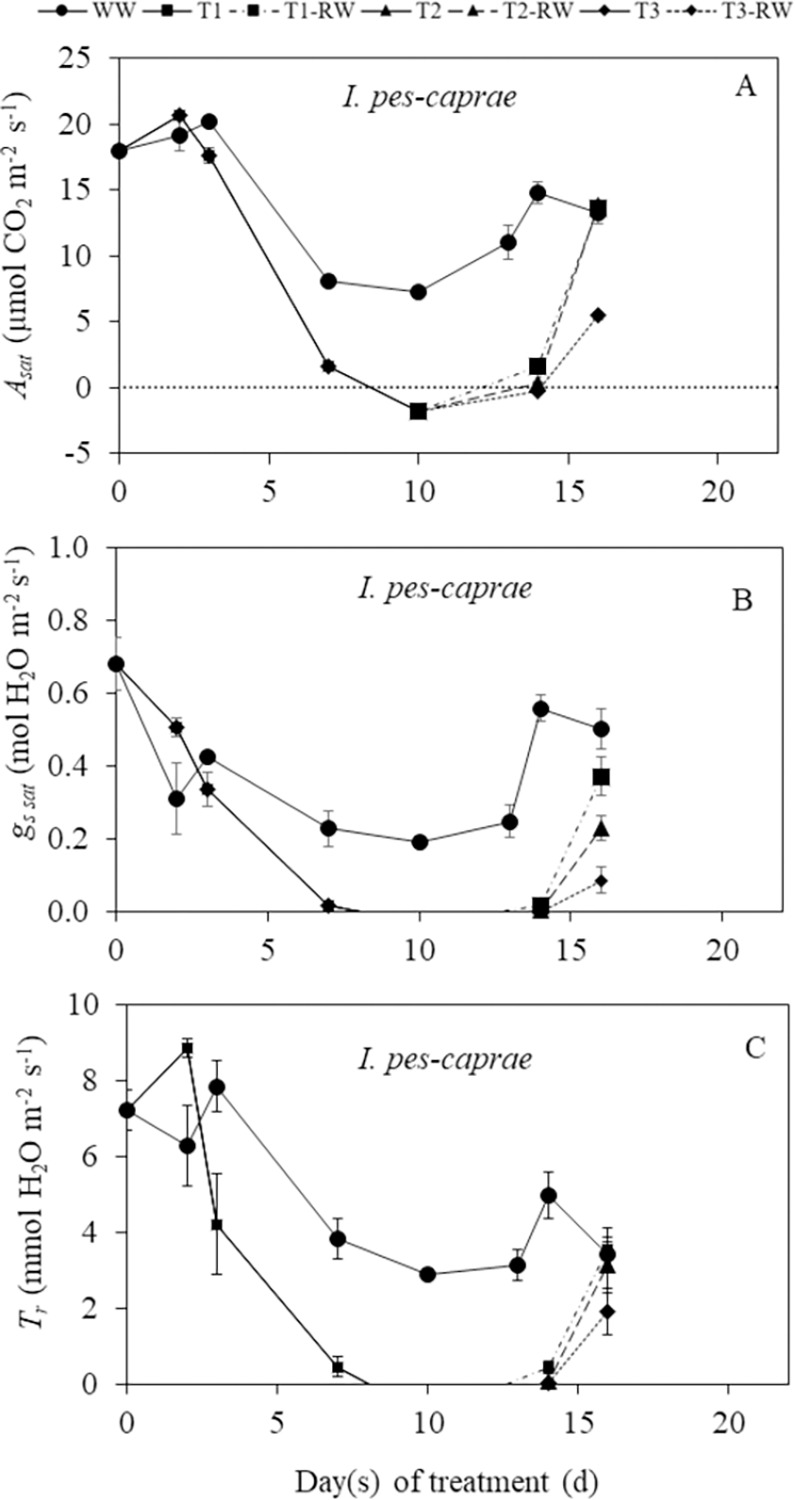
Changes in light-saturated photosynthetic CO_2_ assimilation rate *A*_*sat*_, (A), stomatal conductance, *g*_*s sat*_ (B) and transpiration rate, *T*_*r*_ (C) of *I*. *pes-caprae* during drought stress (DS) and re-watering (RW) treatments. Values are means (± S.E.) (n = 4). Any significant differences (p<0.05) are indicated in the text due to limited space in the figure. WW, well-watered; T1, mild stress; T2, intermediate stress; T3, severe stress.

**Fig 6:**
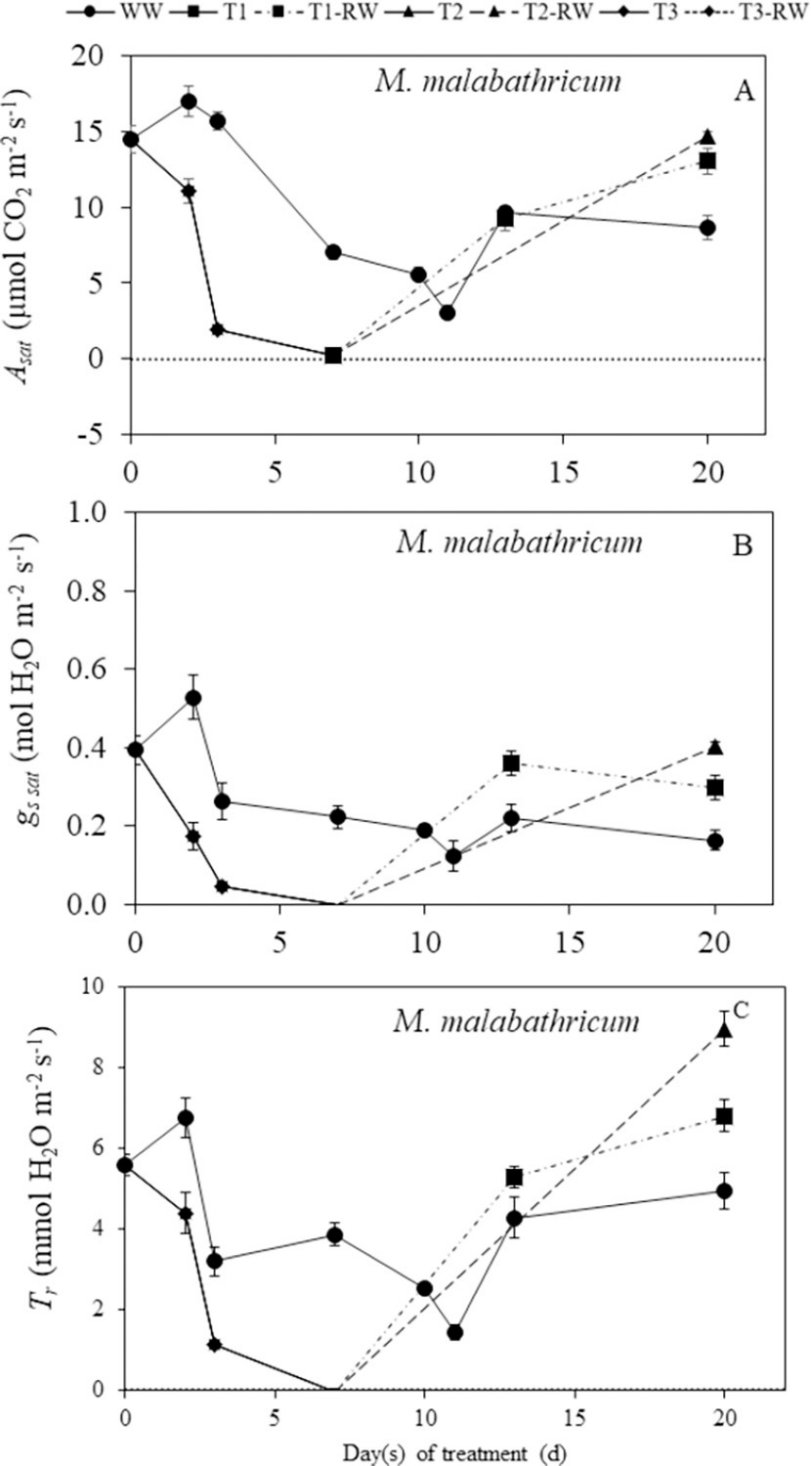
Changes in light-saturated photosynthetic CO_2_ assimilation rate *A*_*sat*_, (A), stomatal conductance, *g*_*s sat*_ (B) and transpiration rate, *T*_*r*_ (C) of *M*. *malabathricum* during drought stress (DS) and re-watering (RW) treatments. Values are means (± S.E.) (n = 4). Any significant differences (p<0.05) are indicated in the text due to limited space in the figure. WW, well-watered; T1, mild stress; T2, intermediate stress; T3, severe stress.

In this study, the DS durations for both *C*. *paniculatum* and *M*. *malabathricum* is day 0 to day 10 while it was 0 to 13 days for *I*. *pes-caprae*. There were lower readings of light-saturated CO_2_ assimilation rate (*A*_*sat*_), stomatal conductance (*g*_*s sat*_) and transpiration rate (*T*_*r*_) for the DS plants compared to the well-watered (WW) plants during DS treatmment for all three species (Figs 4–6). Significant decreases (p<0.05) in *A*_*sat*_, *g*_*s sat*_, and *T*_*r*_ below the levels of WW plants were recorded for *C*. *paniculatum* (Fig 4A–4C) and *I*. *pes-caprae* (Fig 5A–5C) after DS treatment for 7 and 10 days. For *M*. *malabathricum*, these three parameters started to decline significantly compared to those of WW plants (p<0.05) from day 2 to day 7 after DS treatment (Fig 6A–6C). There were significant declines in *A*_*sat*_, *g*_*s sat*_ and *T*_*r*_ of DS-treated *M*. *malabathricum* plants compared to their WW plants (p<0.05) on just day 2 of DS treatment (Fig 6A–6C). However, decreases in these parameters compared to those of WW plants were not significant for *C*. *paniculatum* (Fig 4A–4C) and *I*. *pes-caprae* (Fig 5A–5C) on day 2. The photosynthetic gas exchange parameters for *M*. *malabathricum* on day 10 were not measured since *g*_*s sat*_ and *A*_*sat*_ values were almost zero on day 7, indicating that the stomata had closed completely. Similarly, gas exchange parameters of *I*. *pes-caprae* were not measured on day 13 since its stomata had closed on day 10 (Fig 5A–5C). By the end of treatment, based on their leaf wilting symptoms, DS-treated plants were grouped into mild, intermediate, and severe DS plants, respectively, defined as T1, T2 and T3 plants. Following RW, the *A*_*sat*_, *g*_*s sat*_, and *T*_*r*_ of all three species increased, indicating of recovery. There were full recoveries for *A*_*sat*_, *g*_*s sat*_, and *T*_*r*_ for the DS-treated *C*. *paniculatum* plants to the levels of WW plants on day 20, which was 10 days after RW (Fig 4A–4C). For *I*. *pes-caprae*, the *A*_*sat*_ of T1 and T2 plants after RW (defined as T1 RW and T2 RW) recovered completely on day 16 (6 days after RW). However, on the same day, the *A*_*sat*_ of T3 after RW (defined as T3 RW) was still significantly lower than that of WW plants (p<0.05) (Fig 5A–5C). The *A*_*sat*_ of T1 RW and T2 RW for *M*. *malabathricum* plants recovered completely on day 20 (10 days after RW), but T3 RW plants did not manage to recover and all died off. Thus, the photosynthetic gas exchange of T3 RW was not measured (Fig 6A–6C).

**Fig 7:**
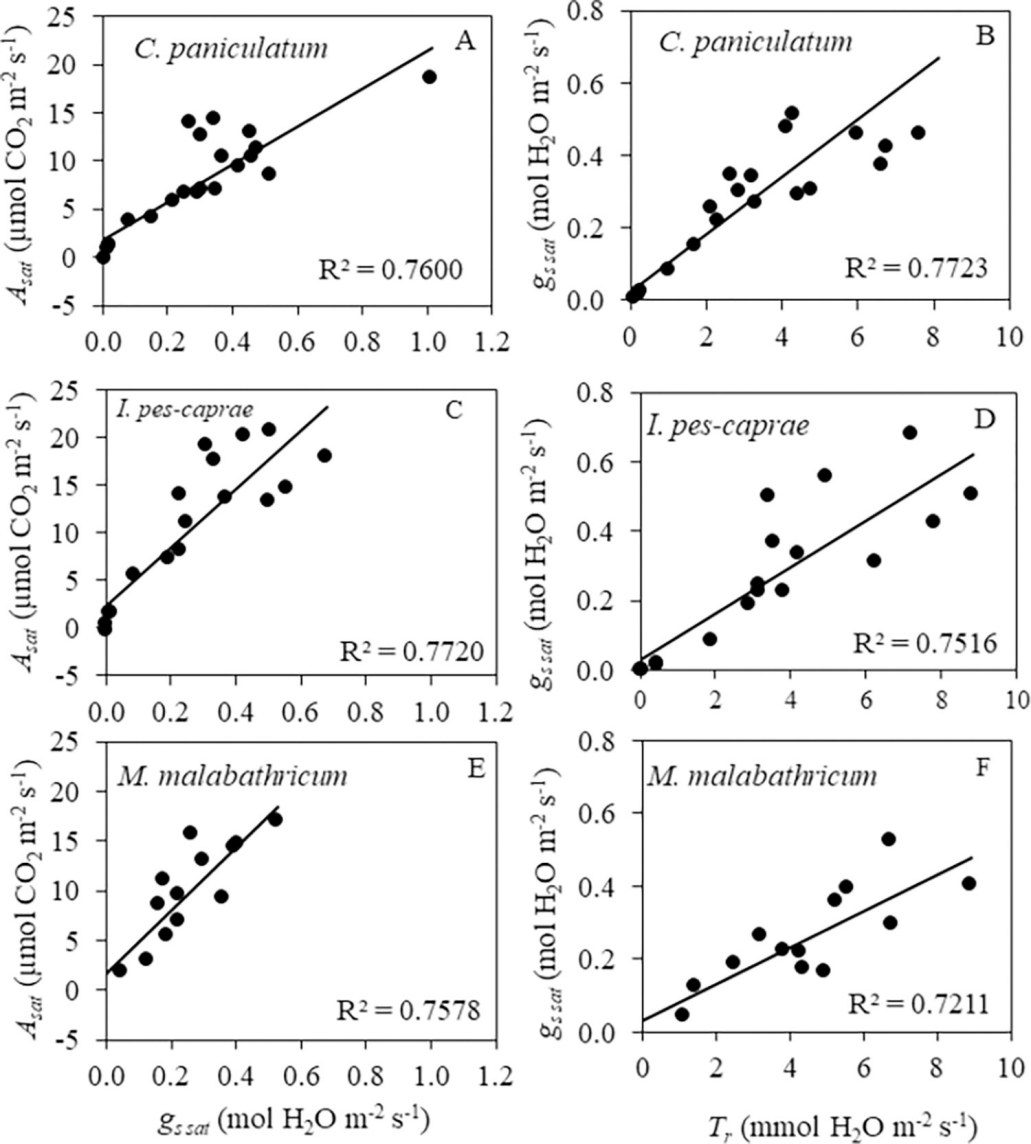
Correlations between *A*_*sat*_ and *g*_*s sat*_ (A, C, E) and *g*_*s sat*_ and *T*_*r*_ (B, D, F) of *C*. *paniculatum* (A, B), *I*. *pes-caprae* (C, D) and *M*. *malabathricum* (E, F) during drought stress (DS) and re-watering (RW) treatment.

There are strong positive linear correlations (R^2^ >0.70) between *A*_*sat*_ and *g*_*s sat*_ (Fig 7A, 7C and 7E) as well as *g*_*s sat*_ and *T*_*r*_ (Fig 7B, 7D and 7F) for all three plant species.

### Changes in leaf relative water content (RWC) and shoot water potential (ψ_shoot_) during DS and RW treatments

**Fig 8:**
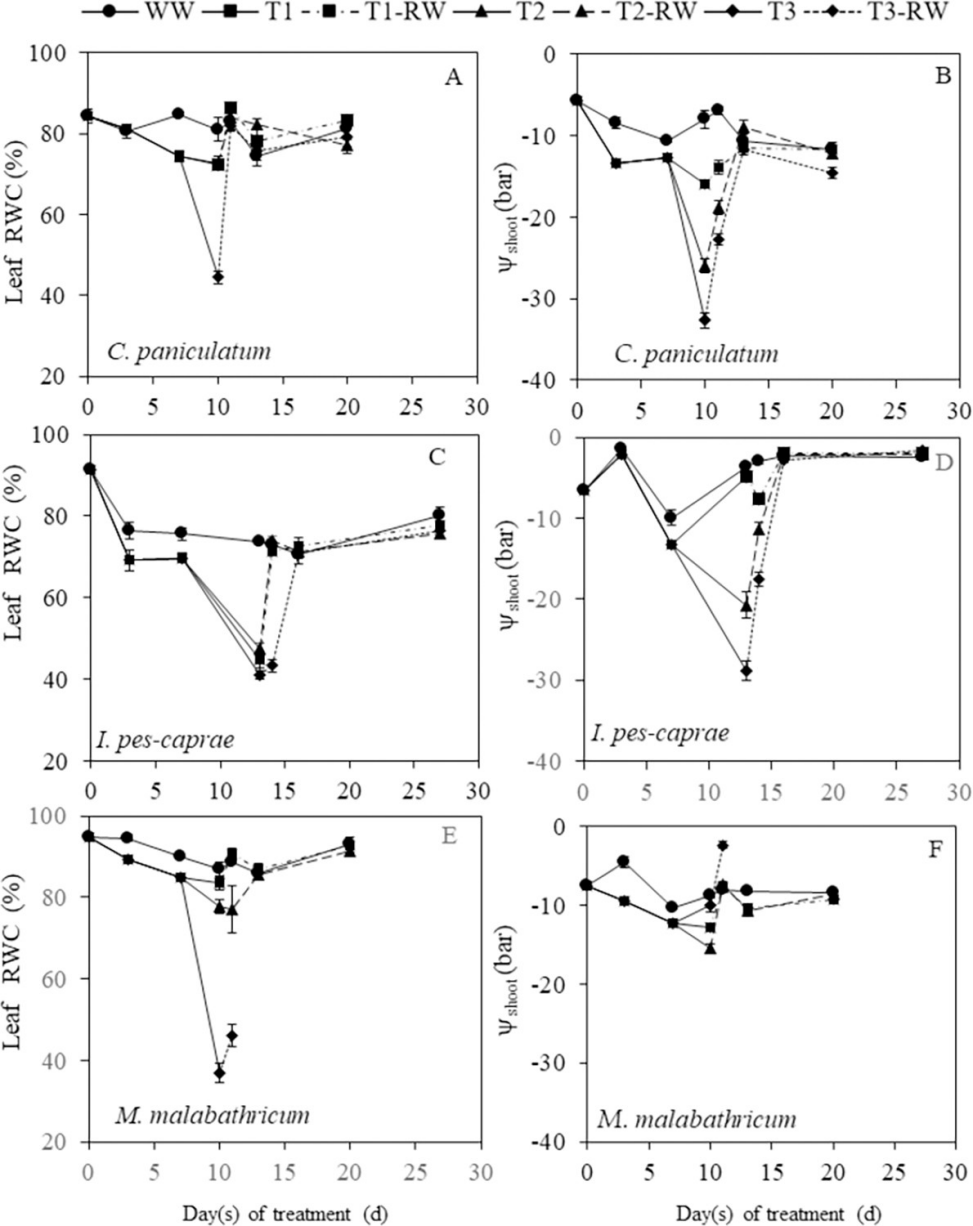
Changes in leaf relative water content, RWC (A, C, E) and shoot water potential, ψ_shoot_, (B, D, F) of *C*. *paniculatum* (A, B), *I*. *pes-caprae* (C, D), and *M*. *malabathricum* (E, F) during drought stress (DS) and re-watering (RW) treatments. Values are means (± S.E.) (n = 4). Any significant differences (p<0.05) are indicated in the text due to limited space in the figure. WW, well-watered; T1, mild stress; T2, intermediate stress; T3, severe stress.

As the DS duration increased from 0 to 10 days for *C*. *paniculatum* and *M*. *malabathricum*, and 0 to 13 days for *I*. *pes-caprae*, leaf RWC and ψ_shoot_ of DS plants decreased compared to corresponding WW plants (Fig 8). On the last day of DS (day 10 after DS treatment), *C*. *paniculatum* grown under T3 conditions had significantly lower leaf RWC compared to WW plants (p<0.05) while T1 and T2 plants maintained similar leaf RWC levels as WW plants (p>0.05, Fig 8A). Comparatively, leaf RWC of *I*. *pes-caprae* grown under T1, T2 and T3 conditions were all significantly lower than corresponding WW plants (p<0.05) (Fig 8C). For *M*. *malabathricum*, only those under the T3 condition had a significantly lower leaf RWC than WW plants (p<0.05) on the last day of DS treatment (day 10 after DS treatment) while leaf RWC of T1 and T2 plants maintained close to levels of WW plants (p>0.05) (Fig 8E). After RW, the leaf RWC for all DS treated *C*. *paniculatum* (Fig 8A) and *I*. *pes-caprae* (Fig 8C) recovered completely. There were no significant differences in leaf RWC between WW and RW *C*. *paniculatum* and *I*. *pes-caprae* plants (p>0,05). However, for *M*. *malabathricum* plants, full recovery of leaf RWC after RW was only observed in those plants under T1 and T2 conditions as *M*. *malabathricum* under T3 conditions continued to dry out and died off after RW (Fig 8E).

ψ_shoot_ of DS plants was significantly lower than that of WW plants for *C*. *paniculatum* and *M*. *malabathricum* on day 3 of DS treatment (Fig 8B and 8F)) but not for *I*. *pes-caprae* (Fig 8D). On the last day of DS (day 10 or 13), the ψ_shoot_ of T1, T2 and T3 plants were significantly lower than those of WW plants for *C*. *paniculatum* (*p*<0.05) (Fig 8B). This was also the case for the T2 and T3 plants for *I*. *pes-caprae* (Fig 8D) and T1 and T2 plants for *M*. *malabathricum* (Fig 8F), reflecting that significant decline in ψ_shoot_ resulted from prolonged DS. After RW, the ψ_shoot_ of DS plants of all three plant species increased to the levels of WW plants and there was no statistical difference between the ψ_shoot_ of DS and WW plants (*p*>0.05), except for the *M*. *malabathricum* T3 plants which did not survive.

The correlations between leaf RWC and leaf gas exchange parameters, and between leaf RWC and ψ_shoot_ are shown in S1 Fig. There were no clear correlations between leaf RWC and *A*_*sat*_ (S1A–S1C Fig), *g*_*s sat*_ (S1D–S1F Fig), or *T*_*r*_ (S1G–S1I Fig) as seen in the low R^2^ values (<0.6). The highest correlation coefficient calculated was R^2^ = 0.5193. Additionally, there was also no clear correlation between leaf RWC and ψ_shoot_ (R^2^<0.5) (S1J–S1L Fig). *M*. *malabathricum* had the poorest correlation between leaf RWC and ψ_shoot_ (R^2^ = 0.0208) (S1L Fig).

### Changes in leaf, stem and root water content

**Fig 9:**
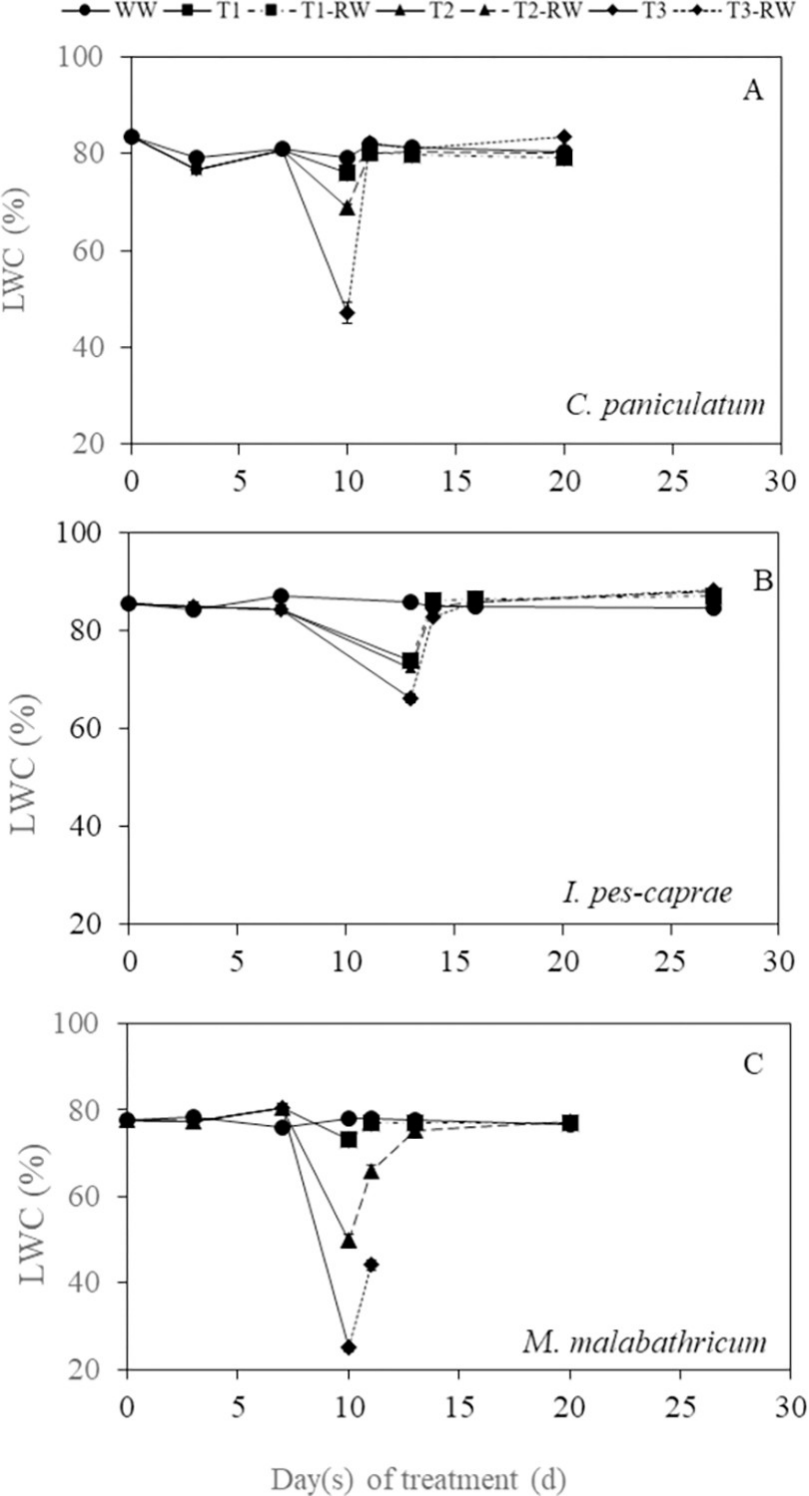
Leaf water content, LWC of *C*. *paniculatum* (A), *I*. *pes-caprae* (B) and *M*. *malabathricum* (C) during drought stress (DS) and re-watering (RW) treatment. Day 10 or 13 was the last day of DS while day 20 or 27 was the last day of RW. Values are means (± S.E.) (n = 4). Significant differences (*p*<0.05) in LWC are indicated in the text due to limited space in the figures.

**Fig 10:**
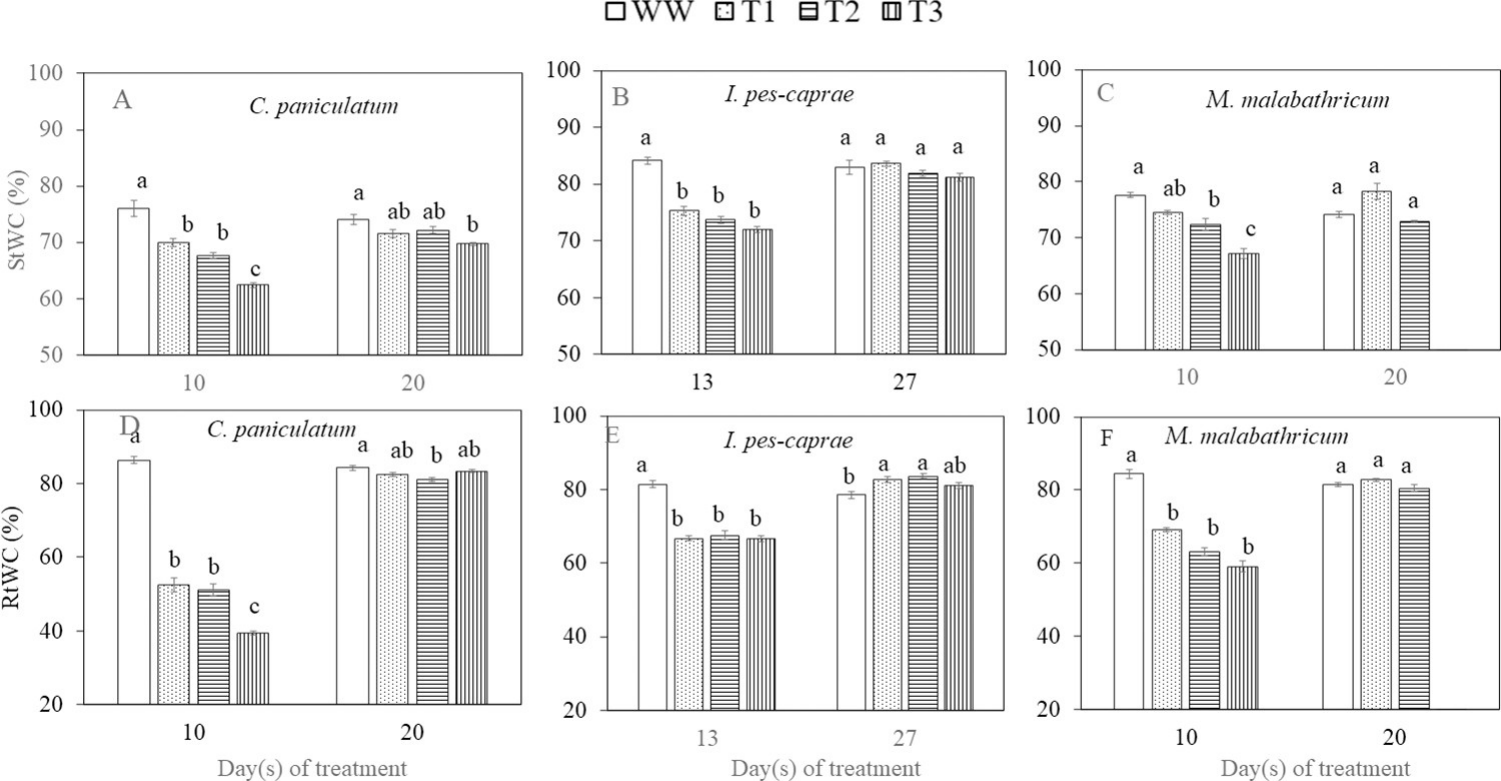
Stem water content, StWC (A-C), and root water content, RtWC (D-F) of *C*. *paniculatum* (A, B), *I*. *pes-caprae* (C, D), and *M*. *malabathricum* (E, F) during drought stress (DS) and re-watering (RW) treatment. Day 10 or 13 was the last day of DS while day 20 or 27 was the last day of RW. Values are means (± S.E.) (n = 4). Means with different letters are statistically different (*p*<0.05) as determined by Tukey’s HSD test for each day. WW, well-watered; T1, mild stress; T2, intermediate stress; T3, severe stress.

Leaf water content (LWC) of the three species decreased as DS duration increased and recovered after RW apart from *M*. *malabathricum* under T3 condition (Fig 9). LWC of T2 and T3 plants were significantly lower than WW plants on the last day of DS treatment (day 10) (p<0.05) for *C*. *paniculatum* (Fig 9A). For *I*. *pes-caprae* (Fig 9B) and *M*. *malabathricum* (Fig 9C), LWC of T1, T2 and T3 plants were all significantly lower compared to corresponding WW plants on the last day of DS treatment which were days 13 and 10 respectively. *M*. *malabathricum* subjected to DS showed the greatest decrease in LWC among the three species, with T3 plants having a 68.1% lower LWC than that of WW plants on day 10 (Fig 9C). For *C*. *paniculatum* and *I*. *pes-caprae* plants, the lowest LWC observed on the last day of DS were, respectively 40.5% (T3) and 22.8% (T3) lower than those of WW plants. Stem water content (StWC) and root water content (RtWC) of all DS-treated plants decreased significantly compared to those of WW plants (Fig 10 apart from the StWC of T1 *M*. *malabathricum* plants on day 10 which was close to WW levels (Fig 10C). The decline in StWC and RtWC was the greatest in *C*. *paniculatum* plants, where StWC of T3 was 18.0% lower (Fig 10A) and RtWC was 54.5% lower than its WW counterpart (Fig 10D). Nonetheless, after 10 or 14 days of RW, the StWC and RtWC of all DS-treated plants recovered to levels close to that of the WW plants (Fig 10), apart from the *M*. *malabathricum* plants grown under T3 condition, which all died due to the severe drought stress. Thus, there were no measurements of LWC, StWC and RtWC on the last day of RW.

### Changes in leaf proline concentration during DS and RW treatments

**Fig 11:**
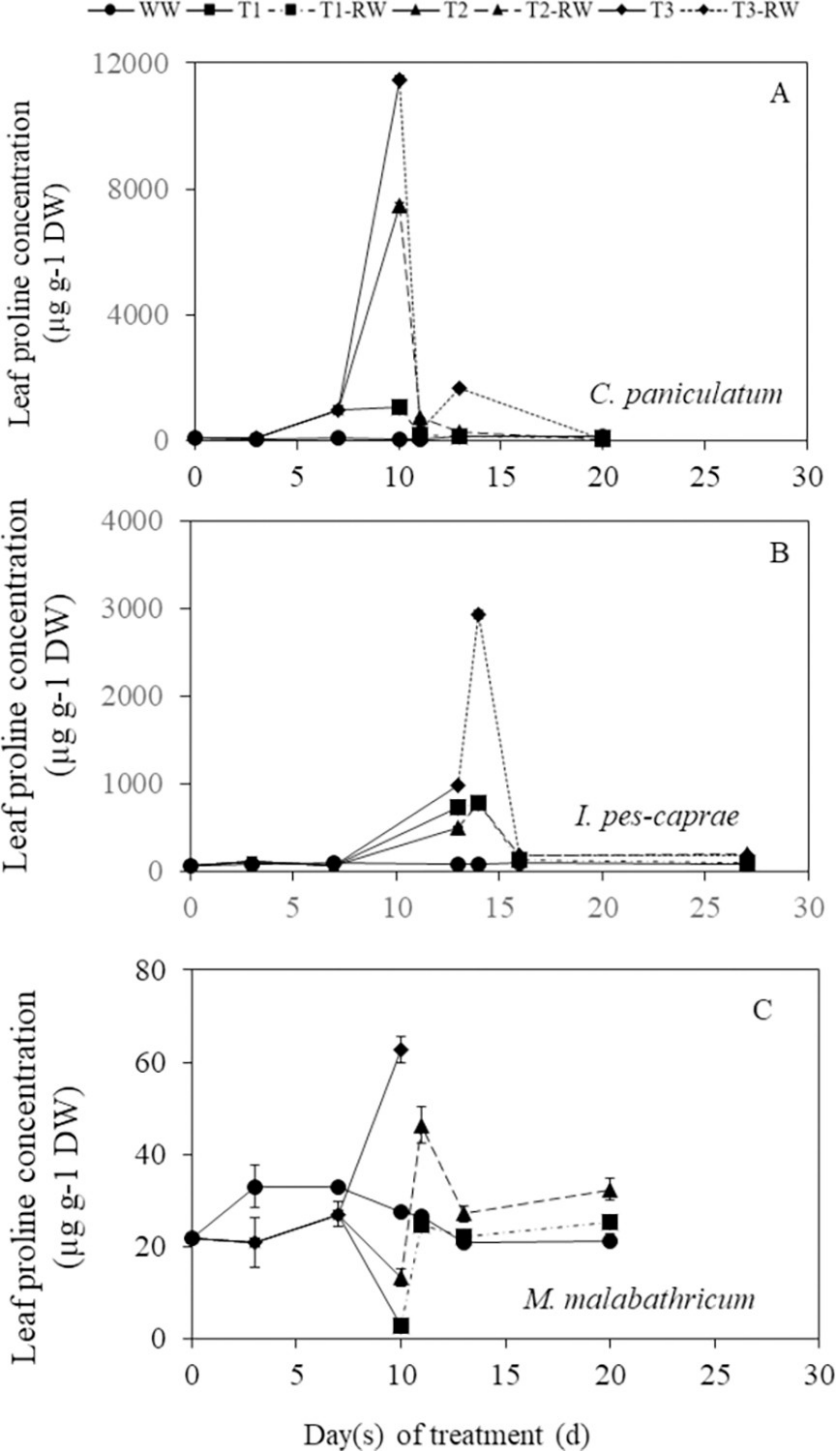
Changes of leaf proline concentration of *C*. *paniculatum* (A), *I*. *pes-caprae* (B) and *M*. *malabathricum* (C) during drought stress (DS) and re-watering (RW) treatment. Values are means (±S.E.) (n = 4). Where there are significant differences (p<0.05) among the means, they are indicated in the text due to the limited space in the figures. WW, well-watered; T1, mild stress; T2, intermediate stress; T3, severe stress.

As the duration of DS increased, leaf proline concentration significantly increased for most DS-treated plants of the three species (*p*<0.05) (Fig 11). Proline accumulation was the highest in *C*. *paniculatum*, peaking at 11469 μg g^-1^ DW in T3 plants on day 10 (Fig 11A). *I*. *pes-caprae* had moderately high proline levels with a maximum of 981.36 μg g^-1^ DW on day 13 (the last day of DS treatment) and continued to increase to 2929 μg g^-1^ DW after RW for one day (day 14 after treatment) (Fig 11B).—Compared to *C*. *paniculatum* and *I*. *pes-caprae* DS plants, proline concentrations were very much lower in *M*. *malabathricum* DS plants, with a maximum of only 62.71 μg g^-1^ DW in T3 plants on day 10 (Fig 11C).

**Fig 12:**
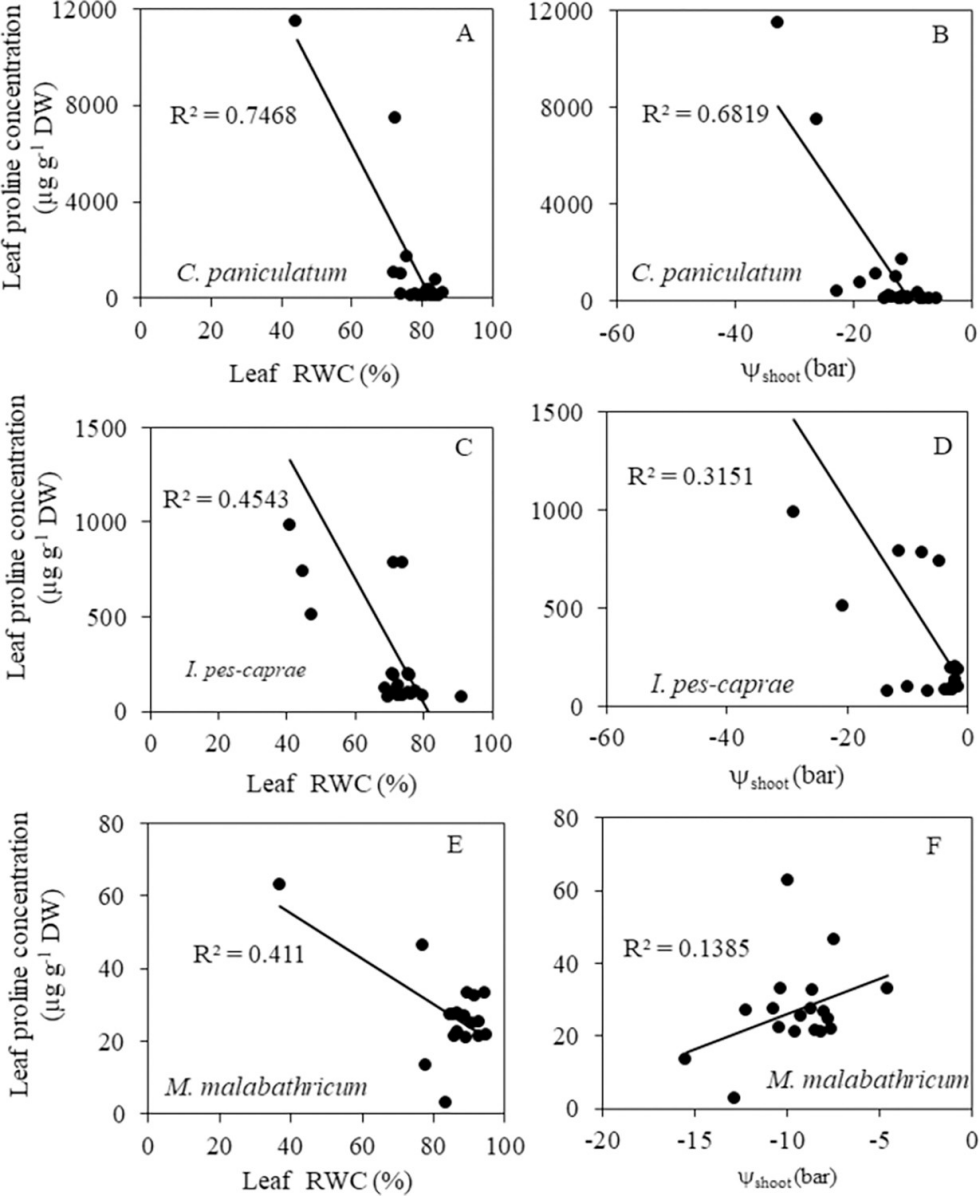
Correlations between leaf proline concentration and leaf RWC (A, C, E) and ψ_shoot_ (B, D, F) of *C*. *paniculatum* (A, B) *I*. *pes-caprae* (C, D) and *M*. *malabathricum* (E, F) during drought stress (DS) and re-watering (RW) treatment.

There is a high level of positive linear correlation between leaf proline concentration and leaf RWC (R^2^ = 0.7468, Fig 12A) and ψ_shoot_ (R^2^ = 0.6819, Fig 12B) for *C*. *paniculatum*. However, correlations between leaf proline concentration and leaf RWC and ψ_shoot_ are poor in *I*. *pes-caprae* (Fig 12C and 12D) and *M*. *malabathricum* plants as reflected by the low R^2^ values (<0.5) (Fig 12E and 12F).

## Discussion

### Responses of photosynthetic gas exchange to DS and RW

It was reported that drought stress decreased photosynthetic CO_2_ assimilation rate compared to well-watered plants [24]. In this study, the decreases in *A*_*sat*_ were observed in all three plant species during DS treatment compared to each of their well-watered counterpart (Figs 4–6). The *g*_*s sat*_ declined similarly during DS treatment, which implies a decrease in stomatal aperture as DS prolonged. As stomatal closure limits the diffusion of CO_2_ into the chloroplasts, this acts as the main driver to slow the photosynthetic CO_2_ assimilation rate [25–28]. Similarly, *T*_*r*_ decreased as DS treatment was prolonged. The reduced *T*_*r*_ is also a result of rapid stomatal closure [10], and this indicates that water loss to the surroundings was reduced. The response to DS differs for the different plant species. The *g*_*s sat*_ of DS-treated *M*. *malabathricum* was significantly lower than corresponding WW plants on day 2 after treatment, while *C*. *paniculatum* and *I*. *pes-caprae* had no significant difference in *g*_*s sat*_ between WW and DS plants on day 2. These results indicate that *M*. *malabathricum* could be an isohydric plant as it closes its stomata early to minimize water loss. However, stomatal closure also results in the suppression of photosynthetic CO_2_ assimilation [12]. On the last day of DS treatment, *g*_*s sat*_ and *A*_*sat*_ values of all three species were close to or below zero. After RW, *A*_*sat*_, *g*_*s sat*_ and *T*_*r*_ of most DS-treated plants increased. *C*. *paniculatum* showed the most recoveries of *A*_*sat*_ (Fig 4A), *g*_*s sat*_ (Fig 4B), and *T*_*r*_ (Fig 4C), on the last day of RW. However, *I*. *pes-caprae* under the T3 condition did not fully reach WW levels (Fig 5). For *M*. *malabathricum* plants, full recoveries were observed for T1 and T2 plants after RW while all T3 plants died before RW (Fig 6) It was also reported that the photosynthetic rate of *Populus nigra* recovered completely from the water deficit despite falling to almost zero during DS treatment [24]. Similar results were also obtained from plants grown in a dry-hot valley savanna of Southwest China [29]. The high level of positive linear correlation (R^2^ > 0.70) between *A*_*sat*_ and *g*_*s sat*_ (Fig 7A, 7C and 7E) shows that there is a relatively strong relationship between these two parameters in all three species. When the stomata close (*g*_*s sat*_ decreases), reduced *A*_*sat*_ would occur. The same correlation was also observed between *g*_*s sat*_ and *T*_*r*_ (Fig 7B, 7D and 7F). When *g*_*s sat*_ decreases, *T*_*r*_ would also decline [30]. Strong stomatal regulation can reduce water loss and help plants survive under prolonged drought stress and recover following re-watering [9, 31].

### Changes in leaf RWC and ψ_shoot_ during DS and RW treatments

The leaf RWC and ψ_shoot_ of all DS-treated plants showed general decreasing trends as DS treatment prolonged for all three species (Fig 8). Leaf RWC reflects not only the degree of leaf hydration but also the balance between water supply to the leaf and transpiration rate [6]. A decrease in leaf RWC of DS-treated plants below 70% indicates a severe leaf water deficit [32]. This was observed in T3 *C*. *paniculatump* plants (Fig 8A) on day 10 of DS treatment, and *I*. *pes-caprae* (T1, T2, T3, Fig 8C) on day 13 of DS treatment, respectively. Leaf RWC of *M*. *malabathricum* was still relatively high except for T3 plants which plunged on the last day of DS (Fig 8E). Leaf RWC of *M*. *malabathricum* T3 plants dropped the most among the three species to a low level of 37% on day 10 (Fig 8E). Severe leaf water deficit causes limitation to CO_2_ uptake due to stomatal closure. With severe water deficits, direct inhibition of photosynthesis occurs (Figs 4–6) [32]. Plant water deficit also leads to a decline in ψ_shoot_ and intracellular water availability, resulting in disturbance of photosynthetic processes and, therefore, affects plant health and plant growth [33]. In this study, on the last day of DS treatment, ψ_shoot_ declined in all three species compared to their WW plants with the least decrease magnitude in *M*. *malabathricum* plants (Fig 8B, 8D and 8F).

In the study with *Hordeum vulgare* and *Triticum aestivum*, Roig-Oliver et al. reported that there were correlations between photosynthesis-related and leaf water-related parameters [34]. However, in this study, poor correlations between leaf RWC and the parameters of photosynthetic gas exchange such as *A*_*sat*_ (**S***1*A–**S***1*C Fig), *g*_*s sat*_ (S1D, S1E and S1F Fig) and *T*_*r*_ (S1G, S1H and S1I Fig) were obtained. For instance, the changes in *A*_*sat*_, *g*_*s sat*_ and *T*_*r*_ of DS-treated *C*. *paniculatum* plants were gradual with less deviation between WW and DS-treated plants (Fig 4) whereas the decrease of leaf RWC for *C*. *paniculatum* was sharp for T3 plants (Fig 8A). For *I*. *pes-caprae*, the decreases in *A*_*sat*_, *g*_*s sat*_ and *T*_*r*_ occurred faster and were more gradual (Fig 5) than the decreases in leaf RWC (Fig 8C) and ψ_shoot_ (Fig 8D). In *M*. *malabathricum*, changes in *A*_*sat*_, *g*_*s sat*_ and *T*_*r*_ during DS and RW were prominent (Fig 6), whereas leaf RWC (Fig 8E) except for T3 plants and ψ_shoot_ (Fig 8F) stayed relatively constant. These inconsistencies in the timing and intensity of the changes in photosynthetic gas exchange and leaf water status contributed to the weak correlation observed. No clear correlation was found between leaf RWC and ψ_shoot_ either (S1J, S1K and S1L Fig).

### Changes in leaf, stem and root water content during DS and RW treatments

Leaf water content (LWC) is a good indicator of plant water status as it is closely linked to soil water availability and plant drought tolerance [35–37]. Leaves are a crucial site for plant-environmental interactions and photosynthesis [37]. Leaf water is a necessary raw material for photosynthesis, which determines plant health [38]. Compared to WW plants, the LWC was significantly lower on the last day of DS treatment for all three species (Fig 9). The decline in LWC of the DS-treated plants below the levels of WW plants suggests that they were unable to efficiently maintain sufficient water in plant organs during DS. The LWC of T2 and T3 *M*. *malabathricum* plants decreased sharply the most across the three species on day 10 after DS treatment (Fig 9C). LWC of *I*. *pes-caprae* DS plants were the least affected (Fig 9B). Upon RW, LWC of all DS-treated plants managed to recover, other than *M*. *malabathricum* T3 plants which were unable to survive. In this study, some plants were also harvested on the last days of DS and RW respectively, to measure their StWC and RtWC. StWC and RtWC of the DS-treated plants were lower than those of WW plants on the last day of DS treatment (Fig 10). Compared to WW plants, decreases in RtWC by the end of DS were generally greater than that of StWC in all three species, suggesting that there was no water uptake by roots from the soil. Apart from the *M*. *malabathricum* plants grown under T3 condition, StWC and RtWC of all DS-treated plants recovered to levels close to that of the WW plants following RW. Re-watering not only enables recovery of different physiological processes and increases leaf and root growth, but also results directly in increased water status within the plant [24]. However, following severe DS treatment (T3), *M*. *malabathricum* was unable to recover after RW. Based on the results of plant water status, *I*. *pes-caprae* seems the most capable of withstanding drought as it tolerated 3 more days of DS treatment compared to the other two species, had a more stable LWC, and an overall higher StWC and RtWC.

### Changes in leaf proline concentration during DS and RW treatments

To defend against leaf water deficit. For example, plants may increase the synthesis of compatible solutes such as proline after being subjected to DS conditions [39–41]. In this study, when leaf RWC and ψ_shoot_ decreased during DS treatment, *C*. *paniculatum* accumulated the highest level of proline (Fig 11A) followed by *I*. *pes-caprae* (Fig 11B), *M*. *malabathricum* had the lowest accumulation of proline (Fig 11C). Accumulating solutes decreases cell osmotic potential, which could be the main factor resulting in the drop of its ψ_shoot_ [14]. In fact, the highest proline concentration recorded was 4 times higher in *C*. *paniculatum* than in *I*. *pes-caprae* and 183 times higher than in *M*. *malabathricum*. This result suggests that *C*. *paniculatum* heavily relies on proline accumulation to cope with DS. This strategy involves accumulating proline into plant cells to decrease osmotic potential, resulting in water being pulled into the cell [14]. *I*. *pes-caprae* also accumulated certain levels of proline during DS and even after one day of RW to tackle DS, but *M*. *malabathricum* was unable to engage this drought stress tolerant mechanism. Various studies align with the increase in proline levels in DS-treated plants [39, 40], and large proline accumulation is usually associated with DS tolerance [41]. It was reported that proline has antioxidant and osmotic properties that improve the drought tolerance of oilseed rape [42]. Thus, *C*. *paniculatum* and *I*. *pes-caprae* are capable of tolerating DS using osmotic adjustment through proline accumulation. There are correlations between leaf RWC and ψ_shoot_ with proline concentration observed in *C*. *paniculatum* (Fig 12A and 12B). However, for *I*. *pes-caprae* and *M*. *malabathricum* plants, the corrections between leaf RWC and ψ_shoot_ with proline concentration were weak due to inconsistencies in the time course changes of these parameters (Fig 12C–12F). In this study, leaf RWC and proline concentration were measured from the same leaves after DS treatment. Furthermore, changes in ψ_shoot_ were much more gradual than changes in proline level. For *I*. *pes-caprae*, DS-treated plants deviated from WW levels on day 13 for leaf RWC, ψ_shoot_ and proline levels, but proline level spiked further on day 14 for T2, whereas leaf RWC and ψ_shoot_ recovered on day 14. Leaf RWC and ψ_shoot_ trends were generally stable for *M*. *malabathricum*, but fluctuations and a decrease in proline on day 10 could partially explain the poor correlations.

### Strategies of three different species to cope with water deficit

Engaging different strategies to cope with water deficits is the product of adaptive traits that enable plant function to changes in water supply as well as recovery when drought stress is relieved [24]. Among the three different tropical perennials, *C*. *paniculatum* has the biggest leaf surface area while the leaf surface area is the smallest for *M*. *malabathricum*. Both C. *paniculatum* and *M*. *malabathricum* are woody shrubs. *I pes-caprae* is an herbaceous creeper with succulent stems. Differences in plant responses to drought stress and re-watering following drought stress have been associated with species depending on their morphological, physiological and biochemical traits [43–45]. For instance, leaf size and even leaf shape, are potential adaptive mechanisms, which may help to limit water loss [43]. Plant resistance to drought is also associated with stem and root morphology [22]. However, this study mainly focused on the changes in physiological and biochemical traits such as photosynthetic gas exchange, leaf RWC, leaf water potential and accumulation of proline after subjecting the three different plant species to DS treatment during the same period in the tropical greenhouse.

Stomatal regulation plays an important role for plants under water deficit. Stomata closure could maintain higher water potential in response to DS. In this study, among the three species, *M*. *malabathricum* seems to be an isohydric plant [11, 46, 47] with tight stomata regulation, which closes its stomata early under drought conditions to prevent water losses (Fig 6B) with a slight decrease in the ψ_shoot_ (Fig 8F). For *M*. *malabathricum*, leaf RWC of DS-treated plants maintained close to the levels of WW plants during DS treatment and only the T3 plant dropped below 70% on the last day (Fig 8E). As a result, the leaf RWC and ψ_shoot_ of *M*. *malabathricum* DS plants stayed relatively stable with a lower accumulation of proline (Fig 11C) throughout the DS treatment compared to the other two species. In comparison, its gas exchange parameters *A*_*sat*_, *g*_*s sat*_ and *T*_*r*_ decreased below the levels of WW plants much earlier on day 2 after DS treatment (Fig 6). This shows that its stomata are very sensitive to DS as the plant actively closes its stomata to minimize water loss and avoid an excessively low leaf RWC. Isohydric plants generally engage a drought-resistant strategy of avoidance to escape from stress. The mechanism of avoidance is characterized by a large root system, a reduction in stomatal conductance, and an increase in leaf thickness to a decrease in leaf area [48]. *M*. *malabathricum* used in this study seems to be marked by these features compared to the other two species. The other drought-resistant mechanism is tolerance through osmotic adjustments [49]. Although decreases of *A*_*sat*_ and *g*_*s sat*_ in *C*. *paniculatum* and *I*. *pes-caprae* occurred a few days later than those of *M*. *malabathricum* (Figs 4 and 5), these two parameters declined gradually from day 7 after DS treatment, suggesting that stomata response to DS treatment prior to the decline in plant water status (Figs 8–10). The sharp increase in proline concentration was observed in both *C*. *paniculatum* and *I*. *pes-caprae* after subjecting to DS treatment with a much higher concentration measured from *C*. *paniculatum*. Accumulation of high level of proline started earlier under T2 and T3 conditions (7 days after DS treatment) in *C*. *paniculatum* than in *I*. *pes-caprae* (day 13 after DS treatment) (Fig 11A and 11B). This result explains why both leaf RWC and ψ_shoot_ of DS-treated *I*. *pes-caprae* were similar to the level of WW plants up till day 7, then decreased sharply to levels significantly lower than WW plants on day 13 (Fig 8C and 8D). It seems that *C*. *paniculatum* and *I*. *pes-caprae* engaged first in an avoidance mechanism and then followed by a tolerant mechanism via osmotic adjustment to cope with drought stress [48, 49]. *C*. *paniculatum* and *I*. *pes-caprae* exhibit anisohydric behaviour where stomatal closure was not so effective in regulating ψ_shoot_ [46, 47]. Apart from stomatal limitation, leaf water deficit also results in non-stomatal limitation such as photodamage in both photosystem I and photosystem II, resulting in the inhibition of electron transfer along the thylakoid electron transport chains. Non-stomatal limitation of photosynthesis causes the decline of *A*_*sat*_ and photosynthetic light use efficiency [50–52]. Non-stomatal limitations of photosynthesis of different tropical perennials under water-deficient conditions are currently being studied by our research team.

After RW, all DS-treated *C*. *paniculatum* and *I*. *pes-caprae* plants maintained the capacity to recover fully for all parameters studied and started to produce new leaves. However, the severe stressed *M*. *malabathricum* plants (T3 condition) did not survive and died off after 10 days of re-watering. Under natural conditions, there is always rain between drought spells. Engaging different strategies to cope with water deficits by plants is not only the mechanism to cope with water deficit but also the recovery when the water deficit is relieved [24]. Using *M*. *malabathricum* plants, Aimee and Normaniza [53] studied the effects of different slope orientations with different levels and directions of light on the physiological performance and erosion rate of the slope soils in Malaysia. They reported that plant growth and physiological performance measured by photosynthetic gas exchange as well as alleviating soil erosion rate of slope soil depends on the slope orientation. However, based on the results of this study, *M*. *malabathricum* may not be suitable to be grown on natural slopes in Singapore as it is the most susceptible species to drought stress and was unable to recover after experiencing severe DS following RW. Nonetheless, *C*. *paniculatum* and *I*. *pes-caprae* could be good candidate plants to be grown on natural slopes.

## Conclusion

Among the plants studied, *M*. *malabathricum* was found to be the least able to acclimatize to drought stress. Therefore, it may not be a suitable species to grow on slopes as prolonged dry spells could cause the death of this species, even upon rewatering. However, *C*. *paniculatum* and *I*. *pes-caprae* were better at tolerating drought. These two species employed mechanisms such as stomata closure to avoid drought stress and osmotic adjustment through proline accumulation. They were also able to fully recover after RW despite a significant decline in water status during drought stress. The outcome of this study provides a better understanding of plant selection in areas (such as slopes) susceptible to natural drought stress conditions. In future studies, non-stomatal limitations such as photosynthetic enzyme activity and mesophyll limitations can be studied to provide a more comprehensive understanding of the responses of these tropical perennials to drought stress and rewatering following drought stress.

## Supporting information

S1 Fig Correlations between leaf RWC and *A*_*sat*_ (A, B, C), *g*_*s sat*_ (D, E, F), *Tr* (G, H, I), and ψ_shoot_ (J, K, L) of *C*. *paniculatum*, *I*. *pes-caprae* and *M*. *malabathricum* during DS and RW (n = 4).
(TIF)
