# Supplementary figures and images for: Photosynthetic gas exchange, plant water relations and osmotic adjustment of three tropical perennials during drought stress and re-watering
Source: PLoS One. 2024 Feb 28;19(2):e0298908. doi: 10.1371/journal.pone.0298908 (PMC10901313; doi:10.1371/journal.pone.0298908)

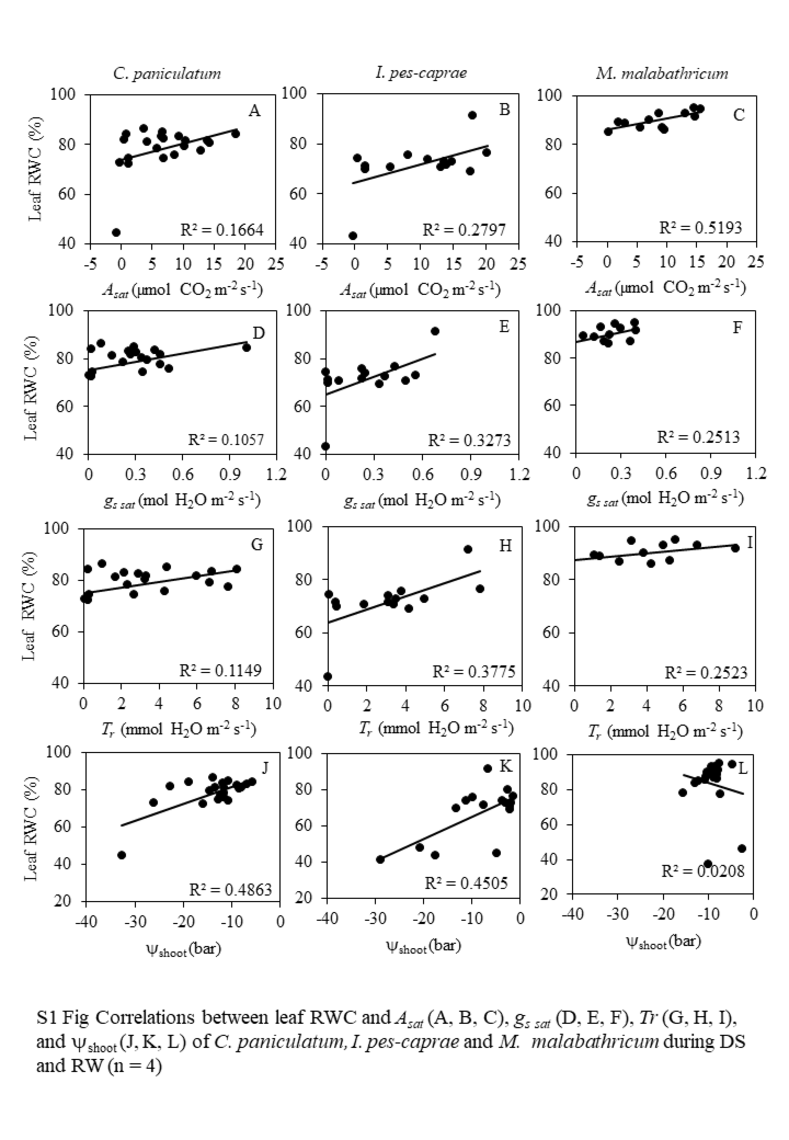

Supplement: S1 Fig — Correlations between leaf RWC and Asat (A, B, C), gs sat (D, E, F), Tr (G, H, I), and ψshoot (J, K, L) of C. paniculatum, I. pes-caprae and M. malabathricum during DS and RW (n = 4). (TIF) [file pone.0298908.s001.tif]
